# Myeloid-derived suppressor cells in hematological malignancies: friends or foes

**DOI:** 10.1186/s13045-019-0797-3

**Published:** 2019-10-22

**Authors:** Meng Lv, Ke Wang, Xiao-jun Huang

**Affiliations:** 1 Peking University People’s Hospital, Peking University Institute of Hematology, National Clinical Research Center for Hematologic Disease, Beijing Key Laboratory of HSCT, No 11 Xizhimen South Street, Beijing, 100044 China; 2 Peking-Tsinghua Center for Life Sciences, Beijing, China

**Keywords:** Myeloid-derived suppressor cells, Hematological malignancies, Lymphoma, Multiple myeloma, Leukemia, Hematopoietic stem cell transplantation

## Abstract

Myeloid-derived suppressor cells (MDSCs) are newly identified immature myeloid cells that are characterized by the ability to suppress immune responses and expand during cancer, infection, and inflammatory diseases. Although MDSCs have attracted a lot of attention in the field of tumor immunology in recent years, little is known about their multiple roles in hematological malignancies as opposed to their roles in solid tumors. This review will help researchers better understand the various characteristics and functions of MDSCs, as well as the potential therapeutic applications of MDSCs in hematological malignancies, including lymphoma, multiple myeloma, leukemia, and hematopoietic stem cell transplantation.

## Background

Myeloid-derived suppressor cells (MDSCs) are a newly identified, heterogeneous population of immature myeloid cells that are characterized by the ability to suppress both innate and adaptive immune responses. The role of MDSCs in solid tumors has been extensively characterized as pro-tumorigenic [1–3]. In intensive clinical studies, circulating and/or infiltrating MDSCs at the tumor site were associated with poor prognosis in patients with solid tumors [4]. Removing MDSCs might contribute to restoring immune surveillance. Meanwhile, conflicting roles have been reported in hematological malignancies [5–10], especially in allogeneic hematopoietic stem cell transplantation (allo-HSCT) for hematological malignancies, which requires the balance between graft-versus-leukemia (GVL) effects and immune tolerance [11]. In this review, we aimed to provide a comprehensive summary of the multiple roles of MDSCs in hematological malignancies and to highlight the double-sided roles of MDSCs.

### What are MDSCs?

In the past 10 years, MDSCs have been defined as a new group of myeloid cells with potent immune regulatory activity. Human MDSCs have been defined as “premature” because of their early-stage cell nature and because of their heterogeneous definitions and their unclear mechanisms of action in human beings. In contrast, the definition of MDSCs in mice is far clearer than in humans; in mice, MDSCs simultaneously express the two markers: CD11b and Gr-1. The expression of Ly-6C and Ly-6G further subdivide murine MDSCs into two different subsets: monocytic-MDSCs (M-MDSCs, CD11b^+^Ly6G^−^Ly6C^high^) and polymorphonuclear or granulocytic-MDSCs (PMN/G-MDSCs, CD11b^+^Ly6G^+^Ly6C^low^) [1, 12]. To mimic these findings in mice, human MDSCs have also been identified by flow cytometry according to cellular markers, but these markers are far from uniform. Human G-MDSCs are defined as CD11b^+^CD15^+^CD14^−^ or CD11b^+^CD14-CD66^+^ cells, as CD15 or CD66b is an activation marker for human granulocytes; however, minimal CD66b is upregulated during nonpathologic conditions. Human M-MDSCs are defined as CD11b^+^CD14^+^HLA-DR^low/−^CD15^−^ cells. CD14 is a typical surface marker for monocyte, while lower or negative HLA-DR help to distinguish M-MDSCs from the mature monocyte and negative CD15 distinguish M-MDSCs from G-MDSCs. The third group of MDSCs was identified as a group of more immature progenitors called Lin- (including CD3, CD14, CD15, CD16, CD19, CD56, HLA-DR-) CD33^+^ cells that are in an early development stage, and it has been proposed that these cells be defined properly as “early-stage MDSCs”(eMDSCs) [12]. In addition to the three main populations, various new definitions of MDSC have been identified in different environments, such as CXCR1^+^CD15^−^CD14^+^HLA-DR^−/low^ [13] PD-L1+ CD11b^+^CD33^+^HLA-DR^−^ [14] MDSC in tumor microenvironments secreted protein acidic and rich in cysteine (SPARC)-positive MDSC in inflammatory state [15], while it remains unknown whether these MDSCs are truly distinct from classical G-MDSCs, M-MDSCs, or eMDSCs.

### How do MDSCs distinguish themselves?

As MDSCs are morphologically and phenotypically similar to neutrophils and monocytes, it is immune suppression that allows MDSCs to be distinguished from other myeloid cell populations. What is so special about these cells that would justify a separate name and what mechanism makes these cells different?

In response to a group of signals produced by tumors or stroma in chronic infection and inflammation, including granulocyte-macrophage colony-stimulating factor (GM-CSF), granulocyte colony-stimulating factor(G-CSF), and macrophage colony-stimulating factor (M-CSF), MDSCs accumulate in more pathological conditions compared with mature neutrophils and monocytes, which are then activated by the second group of signals, including interferon (IFN)-γ, interleukin (IL)-13, IL-4, and IL-6, which finally distinguishes MDSCs based on special gene expression profiles from mature myeloid cells in healthy donors [16]. The endoplasmic reticulum stress response has emerged in recent years as an important mechanism regulating the pathologic activation of MDSCs [17].

With these gene and protein expression profiles, now we know that MDSCs utilize a number of mechanisms to suppress both the innate and adaptive responses of anti-tumor immunity, mostly through the direct inhibition of T cell activation and expansion, including a high level of arginase 1 (ARG1), inducible nitric oxidase (iNOS) [18], or reactive oxygen species (ROS) [19] production, as well as indoleamine 2,3-dioxygenase (2,3-IDO) activity [20]. In addition, MDSCs also mediate immune suppression, including upregulation of regulatory T cells (Tregs) and immune-suppressive cytokines, such as TGF-β and IL-10 [21–24]. Altogether, these unique features of MDSCs allow for their identification and provide insight into their biological activity in clinical disease.

## Are MDSCs always associated with poor outcomes in hematological malignancies?

The role of MDSCs was first established in mouse tumor models. In recent years, the clinical role of MDSCs has emerged, and numerous studies have suggested a positive correlation of MDSCs in peripheral blood or tumor infiltrating sites with high tumor burden, advanced stage tumors, and poor outcomes, which has been intensively reviewed elsewhere [1, 4, 16]. In hematological malignancies, are MDSCs only an enemy?

### Lymphoma

Lymphomas comprise a large group of hematological tumors arising in the lymphatic system, and they share more characteristics with solid tumors than with other hematological malignancies.

In mice model, Serafini et al. demonstrated that MDSCs in a lymphoma animal model shared the same functional properties as MDSCs in solid tumors. By using a murine A20 B cell lymphoma model, a CD45.2^+^A20-HA-GFP tumor was injected intravenously into BALB/c mice. After 28 days, a cell population was found (CD45.2−/GFP^+^) with a phenotype consistent with other murine MDSC phenotypes described in solid tumors. CD45.2−/GFP^+^ cells showed high expression of CD11b, low expression of MHC class I and MHC class II molecules, and expression of Gr1, F4/80, and IL-4Rα, which could inhibit CD8^+^ T cell proliferation and induce pre-existing Tregs. By using arginase and NOS2 inhibitors (NOHA and L-NMMA, respectively), it was shown that arginase-1 and NO were responsible for the inhibition of CD8^+^ T cell proliferation, while only proliferation of CD4^+^ Treg cells was exclusively dependent on arginase-1 [25]. In light of these hopeful results, a series of clinical studies of MDSCs in lymphomas was carried out in the past 10 years.

In peripheral blood of both B cell non-Hodgkin lymphoma(B-NHL) and T-NHL patients, M-MDSCs (CD14^+^HLA-DR^low/−^ ± CD120b^low^) were observed accumulating compared with healthy donors, which were correlated with advanced lymphoma stage, refractory state, higher International Prognostic Index score (IPI), and disease-free survival (DFS). M-MDSCs might return to normal after patients achieve remission. MDSC-dependent T cell suppression was correlated with the upregulated expression of Arg-1, IL-10, programmed death-ligand 1(PD-L1), or S100A12 (a member of the S100 family of calcium-binding proteins involved in T cell suppression through increasing the PD-L1 expression on MDSC). Removing M-MDSCs from patients could restore T cell proliferation [26–32].

G-MDSCs (CD66b^+^CD33dimHLA-DR−) were also demonstrated to accumulate in HL and B-NHL patients compared with healthy donors, while depletion of CD66b^+^ cells could restore T cell proliferation similar to depletion of M-MDSCs [33]. In addition to circulating MDSCs in peripheral blood, Bontkes et al. found that high G-MDSCs (CD11b^+^CD15^+^CD33^int^) in the duodenum are associated with enteropathy-associated T cell lymphoma and its precursor lesions, which may contribute to the development of enteropathy-associated T cell lymphoma (EATL) through the suppression of anti-tumor T cell immunity [34].

In one study of only Hodgkin lymphoma (HL) patients, CD34^+^MDSCs rather than M or G-MDSCs were identified at diagnosis and were found to be the only independent variable for reducing disease-free survival [35]. In extranodal NK/T cell lymphoma (ENKL) patients, total MDSCs (CD33^+^CD11b^+^HLA-DR−) and M-MDSCs were independent prognostic factors for DFS and overall survival (OS). In addition to the elevated signal of arginase-1 and iNOS in MDSCs, IL-17, an inflammatory cytokine produced by CD4^+^ Th17 cells, may promote the induction of MDSCs and enhance the suppressive function of MDSCs on the inhibition of T cell proliferation [36] (Table 1). Regarding the other subtypes of NHL, such as mantle cell lymphoma or follicular lymphoma, no data were found regarding the role of MDSCs.

**Table 1:** MDSCs in lymphoma

Disease cases (*n*)	MDSC subgroups/phenotype definition	Clinical finding	Mechanism/intervention	Year/reference
NHL, *n* = 40	M-MDSCs CD14^+^HLA-DR^low/−^CD120b^low^	Increased M-MDSCs correlated with aggressive disease and suppressed immune functions	Restore T cell proliferation by removing NHL M-MDSC; arginase I↑	2011 [26]
B-NHL, *n* = 42	M-MDSCs CD14^+^ HLA-DR^low/−^	Higher MDSCs vs. healthy donor Higher MDSCs in stage III and IV vs. stage II Higher MDSCs in relapsed/refractory patients	Arginase I↑	2014 [27]
B-NHL, *n* = 22	M-MDSCs CD14^+^ HLA-DR^low/−^	Higher MDSCs with a higher IPI score	IL-10 induced M-MDSCs	2015 [28]
DLBCL, *n* = 66	M-MDSC (CD14 + HLA-DR^Low^) G-MDSC (CD33 + CD11b + Lin-HLA-DR-)	Higher M/G-MDSCs vs. healthy donor M-MDSC number was correlated with the IPI, EFS, and number of circulating Tregs	Upregulated expression of IL-10, S100A12, and PD-L1 attributed to M-MDSC-dependent T cell suppression. T cell proliferation was restored after CD14+ depletion in DLBCL patients.	2016 [29]
T-NHL, *n* = 14	M-MDSCs CD14^+^HLA-DR^low/−^	Higher MDSCs vs. healthy donor	M-MDSCs with PD-L1 expression inhibit T cell proliferation and promote the induction of FoxP3 + Treg	2009 [32]
B cell (HL + NHL), *n* = 124	G/PMN-MDSCs (CD66b^+^CD33^dim^HLA^−^DR^−^ CD11b + CD16+)	Higher MDSCs vs. healthy donor	Restore autologous T proliferation by depletion of CD66b + cells	2016 [33]
Extranodal NK/T cell lymphoma (ENKL), *n* = 32	Total MDSCs HLA-DR^−^CD33^+^CD11b^+^ M (CD14+), G (CD15+)	Higher MDSCs vs. healthy donor Total MDSCs and M-MDSCs were independent predictors for DFS and OS	Higher levels of Arg-1, iNOS, and IL-17; moderate levels of TGFβ and IL-10; but lower levels of CD66b vs. healthy donors, suppressed CD4 but not CD8 activity, inhibited IFNγ but promoted IL-10, IL-17, and TGFβ. Inhibitors of iNOS, Arg-1, and ROS restore T cell proliferation	2015 [36]

In sum, MDSCs, especially M-MDSCs, might contribute to tumorigenesis by inhibiting T cell surveillance in lymphoma, which shares similar mechanisms with solid tumors.

#### Multiple myeloma

Multiple myeloma (MM) is a malignant plasma cell disorder characterized by the accumulation of neoplastic plasma cells in the bone marrow (BM). Immune dysfunction is an important feature of MM patients and leads to infections and increased tumor growth. A variety of immune defects are observed in MM, including cellular abnormalities (e.g., B cells, T cells, and dendritic cells), secretion of immunosuppressive cytokines (e.g., TGF-b, VEGF, and HGF), and increased frequencies of immunosuppressive cell types (including Tregs and MDSCs) [37, 38].

In mice model, it was demonstrated that the percentage of Ly6G^low^ cells is significantly increased in MM-diseased BM compared with naive mice, indicating a skewing of myelopoiesis away from granulopoiesis in the course of MM tumor growth. The higher immunosuppressive activity of MM-derived MDSCs compared with normal MDSCs is accompanied by a higher gene expression of iNOS, Arg-1, and IL-10 [39]. In a similar 5TGM1 model, MDSC expansion in the blood, BM, and spleen could also be observed up to 28 days after MM cell inoculation [40]. In another immunocompetent mouse model, tumor cell lines derived from transgenic Bcl-xl/Myc mice were intravenously injected into syngeneic mice. A clear increased MDSCs (CD11b^+^Gr1^+^) in BM was shown the first week after MM cell inoculation with a similar increase in both M-MDSCs and G-MDSCs subsets. The BM is the primary tumor site for MM cells and is also the site where MDSCs are generated. The direct contact of the cancer cells with myeloid progenitor cells might explain the early MDSC conversion and accumulation [41]. In addition, CD11b^+^Gr1^+^ MDSCs in a mouse model were found contribute to MM chemotherapy resistance [42].

In the peripheral blood of MM patients at diagnosis, M-MDSCs (CD14^+^HLA-DR^low/−^) was first reported to increase compared to healthy donors [43]. Later, it was demonstrated that the level of M-MDSCs was positively correlated with relapsed MM and was negatively related to the treatment response [44]. However, in contrast to the functions of M-MDSCs in lymphoma, G-MDSCs (CD11b^+^CD14-CD33^+^CD15^+^HLA-DR^low^) have been suggested to play a key role in MM pathogenesis. It was reported that G-MDSCs were highly accumulated both in BM and PB in MM patients compared to healthy donors, and this accumulation was also positively associated with the activity of disease in MM [41, 45–48] (Table 2).

**Table 2:** MDSCs in multiple myeloma

Disease cases (*n*)	MDSC subgroups/phenotype definition	Clinical finding	Mechanism/intervention	Year/reference
MM, *n* = 15	G-MDSCs CD11b + CD14-CD33 + CD15+	PB and BM Higher MDSCs vs. healthy donor	S100A9 knockout reduced MDSC accumulation in BM after injection of MM cells	2013 [41]
MM, *n* = 93	M-MDSCs CD14^+^HLA-DR^low/−^	Higher MDSCs diagnosis vs. healthy donor Higher MDSCs in relapsed MM Decreased M-MDSCs after treatment indicated good response	MM cells were able to induce the accumulation of M-MDSCs in vitro, MDSCs induced Treg	2014 [44]
MM, *n* = 17	G-MDSCs CD11b + CD14-CD33 + CD15 + HLA-DR^low^	Higher MDSCs vs. healthy donor associated with the activity of disease in MM	MM cells induced the development of MDSCs from healthy donor peripheral blood mononuclear cells	2013 [45]
MM, *n* = 6	G-MDSCs CD11b + CD14-CD33 + CD15 + HLA-DR^low^	Higher MDSCs in progressive MM vs. healthy donor	MM MDSCs induced the generation of Treg G-CSF increased G-MDSCs	2014 [46]
MM, *n* = 45	G-MDSCs CD11b + CD14-CD33 + CD15 + HLA-DR^low^	MGUS and MM were able to generate the same amount of MDSC, only MM-MSC-educated G-MDSC exhibited suppressive ability	MM G-MDSCs upregulated immune-suppressive factors as ARG1 and TNFalpha, expressed higher levels of PROK2, showed ability to digest bone matrix.	2016 [47]
MM, *n* = 72	G-MDSCs HLA-^DR−/low^/CD33^+^/ CD11b^+^/CD15^+^/CD14−	Higher frequencies of G-MDSCs in both the PB and BM from MM patients, significantly correlated with disease burden by ISS stage.	G-MDSCs enhanced the side population, sphere formation, and expression of cancer stem cell core genes in MM cells. Silencing of piRNA-823 in MM cells reduced the stemness of multiple myeloma stem cells maintained by G-MDSCs	2019 [48]

Similar to other tumor models, in MM patients, Arg-1, iNOS, ROS, and TNF-α were found to be overexpressed by MDSCs [45, 47]. For example, one recent study reported that PMN-MDSCs and their function through increased Arg-1 are associated with MM progression. Arg-1 is mainly expressed by G-MDSCs. PMN-MDSCs and arginase are increased in myeloma and may contribute to resistance to therapy [49]. Tregs could also be induced by MM MDSCs in a cell contact-dependent manner [46]. In addition, MDSCs and MM cells appear to interact in a bidirectional manner, in which MM cells are able to induce MDSCs, probably by mesenchymal stromal cells, and the latter provide a safe haven within the microenvironment for tumor growth and progression [44, 45, 47, 48] (Table 2).

The effect of new therapies, including bortezomib and lenalidomide, on MDSCs are conflicting. In the study by Wang et al., bortezomib combined with dexamethasone resulted in a gradual decrease of the number of MDSCs. When MM cells and PBMCs were cocultured in the presence of bortezomib, a significant decrease in M-MDSCs was observed compared to MM cells and PBMCs alone [44]. However, in the study by Görgün et al., the total number and the immune suppressive capacity of MDSCs did not change after exposure to bortezomib and lenalidomide in vitro [45].

MDSCs, especially G-MDSCs, might interact with MM cells in a bidirectional “win-win” manner, which suggests that treatments targeting M-MDSCs may improve therapeutic outcomes for MM patients.

#### Leukemia and myelodysplastic syndromes

In contrast with lymphoma and MM, studies on MDSCs in leukemia, including acute myeloid leukemia (AML), chronic myeloid leukemia (CML), acute lymphoblastic leukemia (ALL), and chronic lymphocytic leukemia (CLL), and studies on myelodysplastic syndromes (MDS) have been relatively limited.

In mice model of acute leukemia, engraftment of C57BL/6 mice with TIB-49 AML cell lines led to an expansion of CD11b^+^Gr1^+^MDSCs in bone marrow and spleen. Coculture of the AML cell lines or primary AML cells with donor peripheral blood mononuclear cells expanded MDSCs, probably by MUC1-mediated tumor-derived extracellular vehicles [50]. In clinical studies, it has been reported that more eMDSCs (CD11b^+^HLA-DR-CD33^+^Lin-) were accumulated in PB and BM of AML patients when compared with healthy donors [50]. In addition, V-domain Ig suppressor of T cell activation (VISTA), a recently defined negative regulator mediating immune evasion in tumors, was highly expressed on these MDSCs in AML patients; knockdown of VISTA by specific siRNA potently reduced the MDSCs-mediated inhibition of CD8 T cell activity in AML, suggesting a suppressive effect of VISTA on anti-leukemia T cell response [51]. G-MDSC (HLA-DR^−/low^ CD11b^+^CD33^int/high^) was demonstrated to be significantly elevated in both the peripheral blood and BM of patients with B-ALL and was positively correlated with clinical therapeutic responses, such as minimal residual disease [52]. In acute promyelocytic leukemia (APL) patients, tumor-activated ILC2s secreted IL-13 to induce M-MDSCs (CD33^+^CD14^+^HLA-DR-) and to support tumor growth, while ATRA treatment reversed the increase of ILC2-MDSCs in APL [53] (Table 3).

**Table 3:** MDSCs in leukemia and MDS

Disease cases (*n*)	MDSC subgroups/phenotype definition	Clinical finding	Mechanism/intervention	Year/reference
AML, *n* = 8	eMDSCs CD11b + HLA-DR-CD33 + lin-	Higher MDSCs in PB and BM vs. healthy donor MDSCs contribute to tumor-related immune suppression	MUC1 mediates MDSC expansion via the promotion of c-myc expression in secreted extracellular vesicles.	2017 [50]
AML, *n* = 30	MDSCs CD11b + HLA-DR-CD33+	Higher VISTA+ cells among MDSCs from AML patients vs. healthy controls	VISTA knockdown diminished the inhibition of CD8 T cell activity by MDSCs in AML	2018 [51]
ALL-B, *n* = 43	G-MDSCs HLA-DR^−/low^ CD11b + CD33^int/high^	Higher MDSCs in PB and BM vs. healthy donor	B-ALL-derived G-MDSCs was mediated through the production of reactive oxygen species and required direct cell-cell contact, with the potential participation of STAT3 signaling.	2017 [52]
APL, *n* = 31	M-MDSCs CD33 + CD14 + HLA-DR-	Higher MDSCs in PB vs. healthy donor ATRA treatment reverses the increase of ILC2-MDSC in APL	ILC2-derived IL-13 promotes functional M-MDSC	2017 [53]
MDS, *n* = 12	MDSCs Lin–HLA-DR–CD33+	Higher MDSCs in PB vs. healthy donor	Interaction of S100A9 with CD33 promoted MDSCs and induce secretion of IL-10 and TGF-β Early forced maturation of MDSC rescued the hematologic phenotype	2013 [54]
MDS, *n* = 40	eMDSCs CD33(+)HLA-DR(−)Lin(−)	Activation of the CD33 pathway of MDSCs can cause reactive oxygen species (ROS)-induced genomic instability.	Fc-engineered monoclonal antibody against CD33 reduce MDSC, block CD33 downstream signaling preventing immune-suppressive cytokine secretion, and reduced both ROS and the levels of double strand breaks and adducts	2017 [55]
CML, *n* = 36	MDSCs CD11b + CD14 − CD33+	PB MDSC levels were increased in samples from Sokal high-risk patients	Arginase 1↑PD-L1/PD-1 on T cells↑	2013 [56]
CML, *n* = 19	G-MDSCs CD11b + CD33 + CD14-HLADR- M-MDSCs CD14 + HLADR-	PB MDSC levels were increased at diagnosis and returned to normal after therapy	Higher Arg1 expression in MDSCs	2014 [57]
CLL, *n* = 41	M-MDSCs CD14 + HLA-DR^Low^	Higher IDO^hi^ MDSCs in PB vs. healthy donor	CLL cells induce conversion of monocytes into M-MDSCs.	2014 [58]

In the BM of MDS patients, it was demonstrated that more MDSCs (Lin^−^HLA^−^DR–CD33^+^) accumulated compared with healthy donor, the interaction of CD33 with receptor S100A9 (bind to surface glycoprotein receptors on MDSC) promoted MDSCs and induced IL-10 and TGF-β secretion, while early forced maturation of MDSCs rescued the hematologic phenotype of MDS [54]. In addition, CD33-S100A9 initiated suppressive inflammatory signaling cascades that lead to the secretion of ROS, which was strongly correlated to DNA damage and accumulation of phosphorylated γH2AX, a main marker of genomic instability. Fc-engineered monoclonal antibody against CD33 was demonstrated to improves the bone marrow microenvironment by reducing MDSC, blocking CD33 downstream signaling, preventing immune-suppressive cytokine secretion, and reducing both ROS and the levels of double stranded breaks and adducts in MDS [55] (Table 3).

In Sokal high-risk CML patients, Christiansson, L. et al. found that MDSCs (CD11b^+^CD14^−^CD33^+^) and Arg-1 were increased, which upregulated PD-L1 and PD-1 on T cells [56]. Giallongo C. et al. identified that MDSCs (CD11b^+^CD33^+^CD14^−^HLA-DR^−^) were elevated at diagnosis and decreased to normal levels after imatinib therapy in CML patients [57]. In CLL patients, M-MDSCs (CD14^+^HLA-DR^low^ cells) were significantly increased at diagnosis, suppressing in vitro T cell activation and inducing suppressive regulatory T cells. The MDSC-mediated modulation of T cells could be attributed to their increased 2,3-IDO activity. CLL cells induced IDO^hi^ MDSCs from healthy donor monocytes, suggesting bidirectional crosstalk between CLL-cells, MDSCs, and Tregs [58] (Table 3).

As characteristics and pathogenesis of leukemia and MDS are distinct from those of solid tumors, fewer shared mechanisms were found in these hematological malignancies compared with lymphoma and MM, which is in line with the fact that the enhancement of anti-tumor responses by blocking negative immune regulators is a more common mechanism in lymphoma and MM.

#### Hematopoietic stem cell transplantation

In the above models of hematological malignancies, the relationship between MDSCs and the tumor microenvironment is relatively simple, as only bidirectional regulations were involved. In contrast, in the unique model of HSCT for hematological malignancies, a far more complex triangular relationship that correlated the immune balance between GVL effects, graft-versus-host disease (GVHD), and hematological malignancies and MDSCs was noticed [59].

Several mice models have indicated that MDSCs modulate the function of alloreactive T cells and prevent GVHD without impairing the GVL effects. MacDonald first demonstrated MDSCs (CD11b^+^Gr-1^+^), previously described as the granulocyte-monocyte precursor population, could differentiate into class II^+^, CD80/CD86^+^, and CD40^−^ APC during GVHD, which promoted transplant tolerance by MHC class II-restricted generation of IL-10-secreting, Ag-specific regulatory T cells, and more importantly, preserved GVL effects [24]. Messmann reported that MDSCs induced by GM-CSF/G-CSF in vitro inhibited GVHD-induced death and attenuated histologic GVHD by Th2 induction, whereas antitumor cytotoxicity of alloantigen-specific T cells was maintained [60]. Wang reported that adding functional MDSCs to the donor graft alleviated GVHD, whereas removal of MDSCs in vivo exacerbated GVHD. MDSCs from the recipients with GVHD showed much higher suppressive potency compared with those from recipients without GVHD. In addition, MDSC (CD11b^+^Gr-1^+^) accumulation was positively correlated with the severity of GVHD and further increased upon leukemia relapse, suggesting that there are different characteristics of MDSCs in graft and immune reconstitution [61]. Zhang suggested CD115^+^ MDSCs efficiently suppressed GVHD but did not significantly impair GVL effects, as MDSC exhibited cytolytic activities against allogeneic leukemia cells via induced NKG2D^+^ CD8 T cells, while suppressed GVHD by upregulating Tregs [62].

Clinical results have suggested that MDSCs could be expanded by G-CSF mobilization and that MDSCs in grafts are closely associated with lower risk of GVHD in allo-HSCT. Antonio et al. reported that systemic treatment with G-CSF induces the expansion of myeloid cells displaying the M-MDSC phenotype of (Lin^low/neg^HLA-DR^−^CD11b^+^CD33^+^CD14^+^), which is the only graft parameter to predict acute GVHD (aGVHD) [63]. Lv showed that G-CSF induced the expansion of M-MDSCs (Lin^−^HLA^−^DR^−/low^CD33^+^CD11b^+^CD14^+^CD15^dim^CD16^−^) and eMDSCs (Lin^−^HLA-DR^−/low^CD33^+^CD11b^−/low^CD14^−^CD15^−^CD16^−^) in the graft are negatively correlated with the incidence of acute and chronic GVHD without significant influence on relapse and survival [64]. Fan demonstrated that a higher frequency of MDSCs (Lin^low/neg^HLA-DR^−^CD33^+^CD11b^+^) in the G-CSF primed BM than in the G-CSF peripheral blood stem cells harvest (G-PBSC) grafts lead to a better GVHD and relapse-free survival (GRFS) and less GVHD [65]. Wang identified a new subset of eMDSCs (HLA-DR−/lowCD33^+^CD16- cells) in a humanized mice model that may control acute GVHD in mice HSCT, and these cells were also identified as an independent factor that reduced the occurrence of grade II-IV aGvHD in allo-HSCT patients [66]. Schneidawind reported that administration of G-CSF- donor lymphocyte infusion (DLI) results in graft-versus-leukemia effects without exacerbating GVHD because of the M-MDSCs in the DLI component [67].

Hematological malignancy-derived MDSCs exerted negative effects on survival in most circumstances both in mice model and human being, similar to their roles in solid tumors microenvironment. However, in HSCT for hematological malignancies, MDSCs in grafts were regarded as helpful for reducing the risk of GVHD without interfering with the GVL effect for malignancies; meanwhile, the roles of reconstituted MDSCs might still be controversial and remain to be clarified.

## What is behind these disparities?

Considering the disparities of MDSC activity between lymphoma/MM and leukemia/MDS, and in graft or reconstitution in HSCT, one might wonder what mechanisms are behind these disparities?

### Disparities in tumor microenvironment

Take lymphoblastic malignancies as an example. The tumor microenvironment (TME) of lymphoma provides striking examples of a pivotal interaction of hematopoietic tumor cells with stromal cells compared with the TME of acute leukemia. Lymphoma originates and progresses in primary or secondary lymphoid organs where immune cells develop and reside and in which anti-tumor immune responses are typically initiated, suggesting that most are poorly immunogenic and fail to alert innate or adaptive immune sensing mechanisms. In addition, the degree of dependence on MDSCs varies widely among hematologic malignancies. For instance, the normal lymph node architecture is completely replaced by an inflammatory milieu rich in immune suppressive cells in classic HL, which is in contrast to Burkitt lymphoma, where there is a relative paucity of immune suppressive cells, and the normal nodal tissue is almost completely replaced by malignant cells. Thus, the heterogeneous roles of MSCSs could be identified between hematologic malignancies, as previously introduced (Tables 1, 2, and 3) [7].

On the other hand, acute leukemia disseminates rapidly after inception compared with lymphoma and MM, which may negatively impact the initiation and execution of anti-leukemia immunity. Leukemia-specific T cells are never properly activated but, rather, are deleted or anergized upon initial antigen encounter, which is contrary to what is observed in solid tumors and lymphoma, where tumor-specific T cells are primed but become functionally impaired by MDSCs and other components of TMEs [5].

#### Disparities in MDSC origins

The disparity of MDSCs in graft or reconstitution after HSCT might be contributed to the question “how these MDSCs are induced?”. Increasing evidence suggests that G-CSF affects different immune cells [24, 68] that modulate T cell responses either directly by inducing Th2 differentiation [69, 70] or by mobilizing functional regulatory T cells [71] or indirectly through monocytes [72–74], DCs [75], and neutrophils [76]. Thus, nearly all the MDSCs that accumulated in grafts of HSCT or DLIs were induced by G-CSF mobilization rather than by generation by the TME or imbalanced immune environment post-HSCT.

For example, dominated with IL-10/TGF-β signal and Treg induction, MDSCs in G-CSF mobilized grafts were different from the MDSCs cells accumulated during GVHD post-HSCT, which were expressing low pSTAT1 (phosphorylated signal transducer and activator of transcription) and high 2,3-IDO [63]. In addition, MDSC induced by G-CSF mobilization might also be distinct from MDSCs induced in TME of lymphoma, MM, and leukemia, which were characterized by high expression of arginase I, PD-L1, ROS, and STAT-3, etc. (Tables 1, 2, 3, and 4). Therefore, it is worth comparing the differences of MDSCs originated from different models on the single cell level in the future, which could help us to target MDSCs precisely in treatment of hematological malignancies and avoid potential risk of increasing relapse of malignancies post-MDSC-based cellular therapy.

**Table 4:** MDSCs in HSCT

HSCT or models	MDSC subgroups/phenotype definition	Clinical finding	Mechanism/intervention	Year/reference
Unrelated HSCT, *N* = 51	M-MDSCs HLA-DR^low/−^CD14^+^	The frequency of M-MDSCs was significantly increased after allo-HSCT, especially in patients with acute graft-versus-host disease	Blocking the IDO activity of M-MDSCs restore immune tolerance	2013 [20]
Unrelated-HSCT G-PB, *N* = 60	M-MDSCs Lin^low/neg^HLA-DR^−^CD11b^+^CD33^+^CD14^+^	MDSCs in graft as only independent risk factors reducing aGvHD, MDSCs did not impact the relapse rate or the transplant-related mortality rate	Suppress alloreactive T cells	2014 [63]
Haplo-HSCT G-BM and PB, *N* = 62	M-MDSCs Lin-HLA-DR^−/low^CD33 + CD11b + CD14 + CD15dimCD16- eMDSCs Lin^−^HLA-DR^−/low^CD33^+^CD11b^−/low^CD14^−^CD15^−^CD16^−^	MDSCs in graft as independent factors that reduced the occurrence of grade II-IV aGvHD and extensive cGvHD, Delayed M-MDSC reconstitution was associated with aGvHD onset. MDSCs did not impact the relapse rate or the transplant-related mortality rate	Suppress alloreactive T cells	2015 [64]
MSD-HSCT G-PB or G-BM, *N* = 101	MDSCs Lin^low/neg^HLA-DR^−^CD33^+^CD11b^+^	MDSCs in G-BM rather than G-PB was correlated with better GRFS and less GVHD	Immunosuppressive activity of MDSCs was similar in the G-BM and G-PB grafts	2017 [65]
Allo G-BM and PB, *N* = 100	eMDSCs HLA-DR−/lowCD33 + CD16-	MDSCs in G-BM and G-PB as independent factors that reduced the occurrence of grade II-IV aGvHD. MDSCs did not impact the relapse rate or the transplant-related mortality rate	TGF-β signal Th2 differentiation Treg induction	2019 [66]

## Potential application of MDSCs

Considering the disparate roles of MDSCs in non-HSCT and HSCT models of hematological malignancies, it is reasonable to consider MDSCs as a valid therapeutic target in chemotherapy, and even in cellular therapies, since MDSCs contribute to distinct processes in tumor development, progression, and metastasis in the microenvironment [77]. In contrast, MDSCs would be regarded as useful cellular therapy products for prophylaxis or treatment of GVHD post-HSCT.

### MDSC deactivation

Activated MDSCs express high amounts of arginase 1 and NOS2, and inhibitors of both enzymes (L-NMMA for NOS2 and nor NOHA for arginase-1) reversed MDSC suppressive mechanisms in MM and lymphoma models [39, 45, 78]. Consequently, sildenafil (phosphodiesterase-5 inhibitors) treatment of peripheral blood mononuclear cells isolated from MM patients could downregulate arginase 1 and nitric oxide synthase–2 expression, resulted in increased T cell proliferation in vitro [79]. Recently, Borello et al. demonstrated a reduction in Mspike by Tadalafil (phosphodiesterase-5 inhibitors) treatment in an end-stage relapsed/refractory MM patient. BM CD14^+^ cells decreased overtime with Tadalafil treatment. This decrease was associated with a decrease in IL-4Ra, iNOS, and arginase-1 expression in MDSC. BM nitrosylation was also decreased, and T cell activity was enhanced upon Tadalafil administration [80]. In addition, synthetic triterpenoid bardoxolone methyl (CDDO-Me), the activator of the NRF2 transcription factor that results in upregulation of several antioxidant genes, also reduced intracellular ROS and nitrotyrosine levels in EL4 mice, which was accompanied by an increased T cell response and reduced tumor load [81]. Cyclooxygenase-2 (COX2) inhibitors have also been described to reduce MDSC numbers and to have immunosuppressive function in solid tumors, but they have not been tested in hematological malignancies [82, 83]. Targeting IDO1 represents a therapeutic opportunity not only in MDSCs, but also in Treg [84].

#### MDSC depletion

Anti-GR1 antibodies bind with a high affinity to Ly6G molecules and have been extensively used to deplete G-MDSCs in tumor-bearing mice. The effect of anti-GR1 antibodies has been investigated in EL4 tumor-bearing mice, in which a complete elimination of MDSCs in the spleen and peripheral blood was observed [85]. In contrast to anti-GR1 specific antibodies, which predominantly target the granulocytic population, peptibodies were able to deplete both monocytic and granulocytic MDSCs. Intravenous peptibody injection was able to deplete blood, spleen, and intra tumoral MDSCs in distinct lymphoma models (A20, EG7, EL4) and delayed tumor growth in vivo, as determined by tumor size and tumor mass, without inducing effects on other immune cells, including DC and lymphocytes (T, B, and NK cells) [86]. 17-DMAG (HSP90 inhibitors), 5-fluorouracil, and gemcitabine have been explored in a preclinical model of MDSC depletion, with efficacy about 50–75% [87, 88]. Anti-CD33 antibody (gemtuzumab ozogamicin, etc.) have been proved useful in targeting human MDSC which could restore T cell immunity against cancers and enhance CAR-T therapy [55, 89].

#### MDSC differentiation and development

Another mechanism to target the MDSC population is the induction of MDSC differentiation into mature myeloid cells with no suppressive activities. MDSC differentiation can be triggered by distinct vitamins, including vitamin A, vitamin D3, or vitamin E [90–93]. ATRA (all-trans retinoic acid), a vitamin A metabolite, induces the differentiation of monocytic MDSC in DC and macrophages and causes apoptosis of the granulocytic MDSC population. As a consequence, ATRA improved immunotherapy in distinct murine models [93].

S100A8/S100A9 proteins are also involved in MDSC recruitment by the binding of S100 proteins to carboxylated N-glycan receptors. The anti-carboxylated glycan antibody mAbGB3.1 was able to reduce MDSC numbers in the blood and secondary lymphoid organs [94]. Furthermore, mAbGB3.1 was able to block tumor cell proliferation in colorectal cancer [95, 96]. In addition, tasquinimod, a quinoline-3-carboxamide derivative, binds to S100A9 and blocks the interaction with its ligands, receptor of advanced glycation end products (RAGE) and toll-like receptor 4 (TLR4). It has been demonstrated that tasquinimod reduced MDSC accumulation, modulated local tumor immunity, and reduced tumor growth and metastasis [97, 98]. In addition, the N-bisphosphonate zoledronic acid reduced MDSC number and osteoclast formation in MM disease [40]. JAK2/STAT3 inhibitors (JSI-124 and cucurbitacin B) [19, 99] and multikinase inhibitors (sunitinib and sorafenib) were also found to reduce MDSC levels [100, 101].

#### MDSC infusion

In contrast to the role of MDSCs in the chemotherapy of hematological malignancies, MDSCs were found to be beneficial in grafts of HSCT or DLI, which reduced GVHD and preserved the GVL effect [102]. Lim reported in a preclinical model that ex vivo-generated human cord blood MDSCs could attenuate clinical and pathologic cGVHD severity by preserving thymus function and regulating Th17 signaling, suggesting a possible therapeutic strategy for the clinical application of MDSC infusion [103]. Further, caution must be taken in utilizing MDSC infusion due to the conflicting role of reconstituted MDSCs, which might contribute to GVHD onset.

## Conclusions

**Fig. 1:**
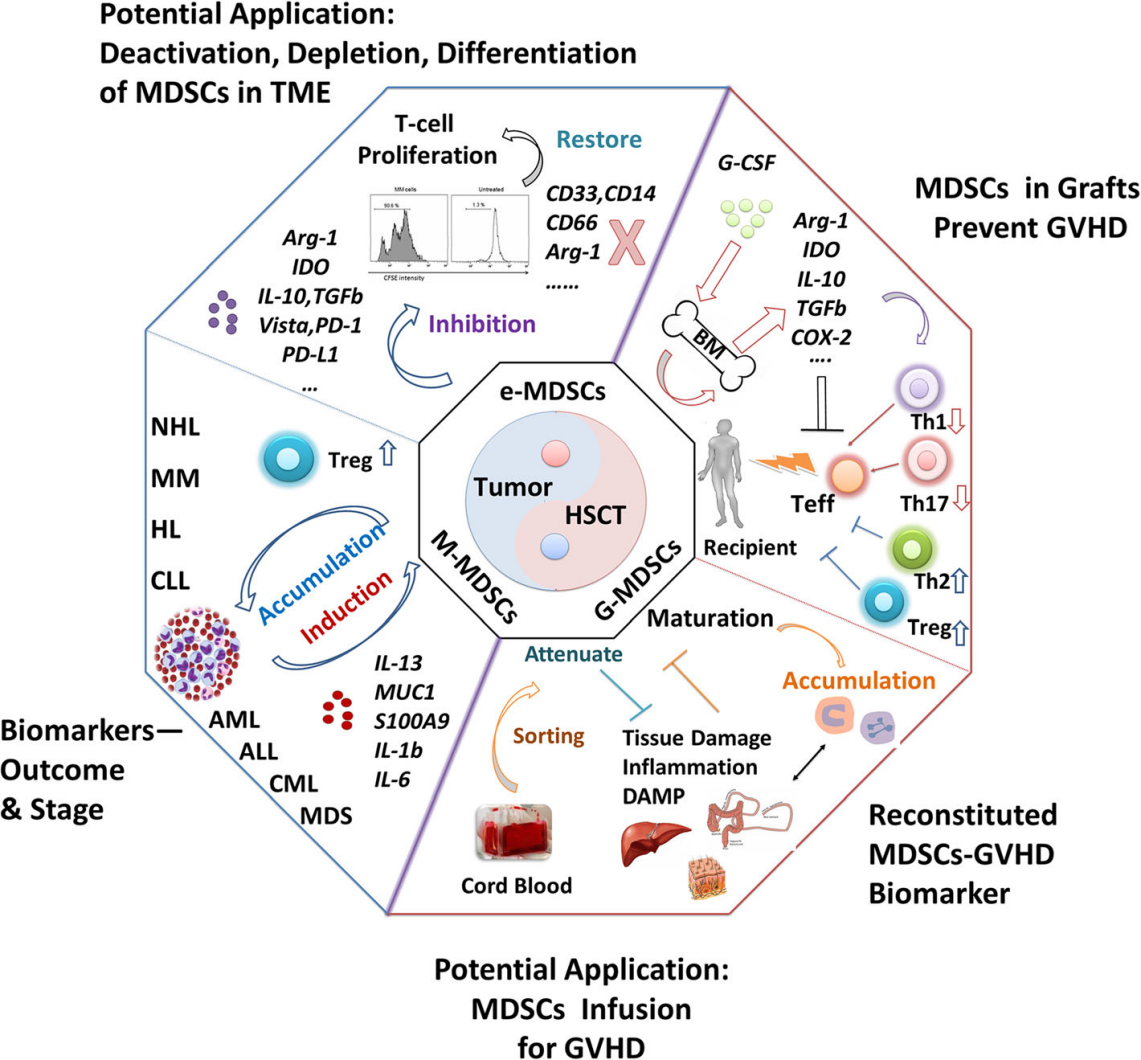
Conflict roles of MDSCs in hematological malignancies

MDSCs are new but important regulators that hamper the host anti-tumor immune response by inhibition of T cell proliferation, cytokine secretion, and recruitment of regulatory T cells in hematological malignancies, similar to their actions in solid tumors, which would be a key target in chemotherapy and immune therapy. On the other hand, transfusing MDSCs would be a potentially beneficial therapy for reducing GVHD but for preserving GVL effect post-allo-HSCT for hematological malignancies. The double-sided roles of MDSCs need to be further clarified in future (Fig. 1).

## Data Availability

All data generated or analyzed during this study are included in this published article and its supplementary information files.

## Abbreviations

2,3-IDO: Indoleamine 2,3-dioxygenase
ALL: Acute lymphoblastic leukemia
Allo-HSCT: Allogeneic hematopoietic stem cell transplantation
AML: Acute myeloid leukemia
APL: Acute promyelocytic leukemia
ARG1: Arginase 1
ATRA: All-trans retinoic acid
BM: Bone marrow
CLL: Chronic lymphocytic leukemia
CML: Chronic myeloid leukemia
COX2: Cyclooxygenase-2
DCs: Dendritic cells
DFS: Disease-free survival
DLI: Donor lymphocyte infusion
EATL: Enteropathy-associated T cell lymphoma
eMDSCs: Early-stage MDSCs
ENKL: Extranodal NK/T cell lymphoma
G-CSF: Granulocyte colony-stimulating factor
GM-CSF: Granulocyte-macrophage colony-stimulating factor
G-PBSC: G-CSF peripheral blood stem cells harvest
GRFS: GVHD and relapse-free survival
GVHD: Graft-versus-host disease
GVL: Graft-versus-leukemia
IFN: Interferon
IL: Interleukin
iNOS: Inducible nitric oxidase
IPI: International Prognostic Index score
M-CSF: Macrophage colony-stimulating factor
MDS: Myelodysplastic syndromes
MDSCs: Myeloid-derived suppressor cells
MM: Multiple myeloma
M-MDSCs: Monocytic-MDSCs
NHL: Non-Hodgkin lymphoma
OS: Overall survival
PD-L1: Programmed death-ligand 1
PMN/G-MDSCs: Polymorphonuclear or granulocytic-MDSCs
RAGE: Receptor of advanced glycation end products
ROS: Reactive oxygen species
TLR4: Toll-like receptor 4
TME: Tumor microenvironment
Tregs: Regulatory T cells
VISTA: V-domain Ig suppressor of T cell activation
